# Shewanella algae – A Novel Organism Causing Bacteremia: A Rare Case and Literature Review

**DOI:** 10.7759/cureus.10676

**Published:** 2020-09-27

**Authors:** Michelle Bernshteyn, Prashanth Ashok Kumar, Sumendra Joshi

**Affiliations:** 1 Internal Medicine, State University of New York (SUNY) Upstate Medical University, Syracuse, USA; 2 Pulmonology and Critical Care, State University of New York (SUNY) Upstate Medical University Hospital, Syracuse, USA

**Keywords:** shewanella algae, antibiotics, sepsis

## Abstract

Shewanella species are distributed ubiquitously in the soil and water, being common in the marine habitat. Although these bacilli were thought to be rarely pathogenic, there has been an increasing number of reports of them being implicated in a wide variety of clinically significant infections. Three distinct species were initially recognized by MacDonell and Colwell. They were Shewanella putrefaciens, hanedai and benthica. Shewanella algae, which is the most common human clinical isolate, was believed to be a strain of Shewanella putrefaciens by some authors, and was later grouped as a separate and distinct entity. With multi-drug resistance on the rise and the lack of large-scale systemic studies, we describe a case of bacteremia caused by this rare organism. We hope to increase the awareness among care providers on the same.

## Introduction

Shewanella species are a group of saprophytic gram negative, motile bacilli, that are considered an emerging human pathogen [1, 2]. It was in 1931, that this bacterium was first isolated from putrefied butter [3]. Despite the presence of more than 30 different species, only Shewanella putrefaciens, and more so Shewanella algae, have been implicated in clinically relevant infections [1]. Shewanella cases have been reported worldwide but are more common in the tropics and during summer months in temperate zones [4].

## Case presentation

**Figure 1:**
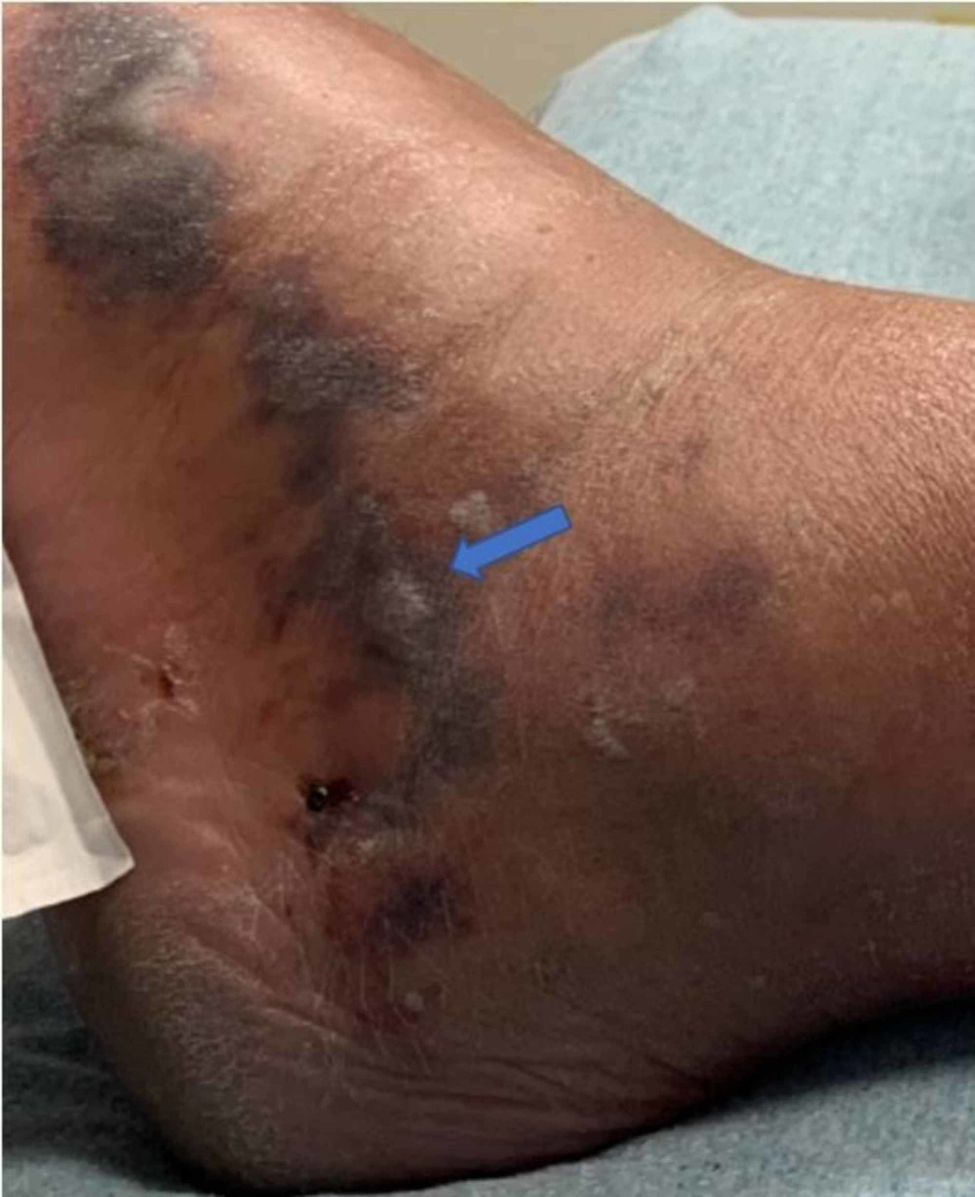
Left lower extremity can be visualized in this figure. Swelling and tenderness were present over the dorsum of the foot and extending to the lateral ankle. Hemorrhagic bullae were present, as indicated by the blue arrow.

A 58-year-old male with a history of atopic dermatitis, psoriasis on risankizumab, and hypertension presented with left lower extremity swelling. Of note, the patient was prescribed 150 milligrams of risankizumab every three months via subcutaneous injection. He had been on this medication for a total of six months. It was discontinued on presentation. He recently went on vacation and noticed a bite in his left foot after walking barefoot on the beach. Shortly after, he felt a burning sensation and experienced swelling. This did not affect ambulation. Due to the persistent swelling and pain in the limb, the patient decided to come to the hospital (Figure 1).

An ultrasound demonstrated no acute pathological changes. The patient was discharged on a course of cephalexin, however, was recalled after the blood cultures grew gram negative rods. He developed severe sepsis with acute kidney injury and lactic acidosis. His creatinine was 1.7 mg/dL, white blood cell count of 11,300/uL, and lactate of 2.8 mmol/L. The patient was started on cefepime and vancomycin. Computed tomography (CT) of left lower extremity demonstrated diffuse predominantly superficial cutaneous and subcutaneous soft tissue stranding. This imaging study was performed to assess the extent of disease and possible involvement of other structures. Necrotizing fasciitis could not be excluded. There was no abscess seen. The patient was switched to piperacillin-tazobactam and admitted to the intensive care unit for closer monitoring. The following day, he developed hemorrhagic bullae and progressive swelling that involved his ankle joint. Incision and drainage of the swelling had to be done. Once sensitivities were available, antibiotics were changed to ceftazidime and ciprofloxacin. Both of the initial blood cultures and wound culture from debridement had resulted, both of which grew Shewanella algae in aerobic and anaerobic broths. The patient was discharged on fourteen days of oral ciprofloxacin with surgical follow-up. Although, he recovered from the current infection, his recovery was characterized by recurrent soft tissue infection of the same limb requiring hospitalization, broad spectrum antibiotic treatment and surgical interventions. Cultures demonstrated Methicillin-resistant Staphylococcus aureus (MRSA). The patient underwent incision and drainage procedure and imaging of the extremity could not confidently rule out osteomyelitis. Therefore, he completed a six-week course of intravenous vancomycin and oral ciprofloxacin. The latter was replaced with oral doxycycline during the treatment course due to elevated inflammatory markers and acute kidney injury. The patient was reported to be asymptomatic for four months. He presented to the outpatient infectious disease office with increased pain of the left lower extremity. Magnetic resonance imaging (MRI) demonstrated soft tissue swelling consistent with cellulitis, without evidence of osteomyelitis or abscess. Given his history of MRSA infection, the patient was prescribed a four-week course of doxycycline, bringing us to present day.

## Discussion

Shewanella algae is believed to carry a higher pathogenic potential. Production of hemolysin has been associated with the same. 16s rRNA gene sequencing analysis is used for identifying Shewanella [5]. The differences in the molecular percentage of G and C and biochemical properties are used to distinguish Shewanella putrefaciens and algae [2]. Many labs in the past were not able to distinguish Shewanella putrefaciens and algae. Over the past decade, the use of matrix-assisted laser desorption/ionization time-of-flight mass spectrometry has enabled detection of the latter and thus we see an increasing number of infections caused by Shewanella algae [6].

Vignier et al. in 2013 published an extensive review of all the cases of Shewanella that were published until then. They found 239 prior cases in 56 reported studies and reviewed a total of 260 cases. Most cases were from Europe (40%), though worldwide distribution was noted. 11% of the patients were immunocompromised. The skin and mucosa as port of entry was implicated in 53% of cases with the other cases having no known entry point. On analyzing the clinical spectrum, skin (27%) and ear infections (33%) were the most common. The spectrum of pathology was wide and included abdominal and biliary tract infections (17%) as well as respiratory infections (13%). Bone, urinary tract, and eye infections, endocarditis, meningitis, and cerebral abscess were also reported in small numbers. Bacteremia was a common complication (28%). Mortality stood at 13% out of 108 patients [1]. Skin blistering, as seen in our patient, has been reported in a few cases [2]. The spectrum of skin pathology is also diverse and ranges from cellulitis to necrotizing fasciitis [1]. Nath et al. reported two cases of gastroenteritis caused by Shewanella algae [5]. There have been more cases of the bacterium colonizing medical equipment and its increasing involvement in hospital-based infections poses a new challenge [2].

Most patients are managed successfully with surgical therapy, drainage, and antibiotics. Pre-existing risk factors like peripheral vascular disease, peripheral neuropathy, conditions causing edema, and stasis, like chronic liver disease and heart failure, may cause complications and affect outcomes [7, 8]. Beta lactamases like amoxicillin, third generation cephalosporins, and piperacillin-tazobactam provide good coverage. Ciprofloxacin and Gentamycin are also effective [1]. There are reports which have demonstrated resistance to Imipenem and Meropenem, most likely due to oxacillinase production. Resistance to piperacillin-tazobactam has also been noted in some cases [1, 2, 9].

Of note, the patient has been taking risankizumab for underlying psoriasis. This medication is an interleukin-23 inhibitor which prevents the release of cytokines, decreasing overall inflammation [10]. Two 52-week phase 3 trials demonstrated more frequent infections in the risankizumab groups as compared to the placebo groups. It was noted that the most common infection reported was an upper respiratory tract infection. Infections classified as serious demonstrated no significant difference between the groups [11].

## Conclusions

We thus conclude that it is imperative to consider Shewanella infection in the context of marine or aquatic exposure, especially during relatively warmer months. Given the increasing incidence of this rare pathogen and the emergence of drug resistance, further research for effective and judicious use of antimicrobials is needed.
